# How to Define Psychological Therapy and Psychotherapy? – An Interdisciplinary Proposal

**DOI:** 10.32872/cpe.22829

**Published:** 2026-05-29

**Authors:** Winfried Rief, Julia Asbrand, Luisa Baumgärtner, Ulrike Dinger, Christoph Flückiger, Nina Heinrichs, Sabine C. Herpertz, Tania Lincoln, Silke Lipinski, Wolfgang Lutz, Bernhard Strauss, Svenja Taubner, Oliver Vorthmann, Jan Philipp Klein

**Affiliations:** 1 Division of Clinical Psychology and Psychotherapy, University of Marburg, Marburg, Germany; 2 Clinical Psychology in Childhood and Adolescence, University of Jena, Jena, Germany; 3 Wilhelm Wundt Institute of Psychology, University of Leipzig, Leipzig, Germany; 4 Clinical Institute for Psychosomatic Medicine and Psychotherapy, Medical Faculty and University Hospital Düsseldorf, Düsseldorf, Germany; 5 Clinical Psychology II, University of Kassel, Kassel, Germany; 6 Clinical Psychology in Children and Adolescents, Bielefeld University, Bielefeld, Germany; 7 Department of Psychiatry and Psychotherapy, University of Heidelberg, Heidelberg, Germany; 8 German Center for Mental Health (DZPG), Site Mannheim/Heidelberg/Ulm, Germany; 9 Clinical Psychology and Psychotherapy, University of Hamburg, Hamburg, Germany; 10 Trialogical Board of the German Center for Mental Health (DZPG), Berlin-Potsdam Site, Germany; 11 Clinical Psychology of Social Interaction, Humboldt-Universität zu Berlin, Berlin, Germany; 12 Clinical Psychology and Psychotherapy, Trier University, Trier, Germany; 13 Institute of Psychosocial Medicine, Psychotherapy and Psychooncology, University Hospital Jena, Jena, Germany; 14 Institute for Psychosocial Prevention and Psychotherapy, University Hospital of Heidelberg, Heidelberg, Germany; 15 DepressionsLiga, Berlin, Germany; 16 Department of Psychiatry, Psychosomatics and Psychotherapy, University of Lübeck, Lübeck, Germany

**Keywords:** psychotherapy, definition, evidence-based treatments, transtheoretical approach, integration

## Abstract

**Background:**

The definition of psychological treatments and psychotherapy has various implications for communication within research and clinical care, as well as for legal issues — particularly in countries with psychotherapy acts. It can define what should be covered by public healthcare systems and who should be permitted to provide these services.

**Method:**

We assembled a group of psychotherapists in Germany to develop an inclusive, scientific definition of psychotherapy. This definition should serve as an umbrella that is not limited to traditional treatment schools and is suitable for science, clinical care and legal acts. The group comprised people with different therapeutic backgrounds (e.g., CBT, psychodynamic and systemic therapy), experts from various disciplines (e.g., psychology, psychiatry and psychosomatic medicine), people focusing on different age groups (e.g., adults and youth), individuals with lived experience of psychotherapy and early career clinicians.

**Results:**

A literature review led us to consider four aspects of a definition. What are the treatment means or 'tools'? (modality of treatment); how does the treatment work? (Proposed change mechanisms, assumptions about causal and evidence-based processes and concepts); who receives psychotherapy? (target group); and who provides psychotherapy? (Provider). Based on these aspects, we offer a definition of psychological therapy.

**Discussion:**

Our suggested definition is not bound to specific treatment orientations, but is intended to encompass both traditional approaches and innovative developments. Our aim is to promote the evidence-based provision of psychological therapy within healthcare systems and legal frameworks.

## Highlights

- A definition of psychological therapy/psychotherapy should address aspects such as treatment modality, processes and mechanisms of change, receiver and provider of treatments.
- We provide a suggestion for a definition of psychotherapy that is also suitable for legal regulations.

The definition of psychological treatments and psychotherapy has various implications, and should offer a framework for categorization within research and clinical care. Even more importantly, the definition of psychotherapy can have legal relevance, in particular in countries with psychotherapy acts. A psychotherapy act defines the role of psychological therapy in healthcare systems (including reimbursement) and who is permitted to provide them. In many countries, a need for legal regulations of psychological therapy was expressed (e.g., Flora, 2024). Therefore, the definition of psychological therapy has an important practical value, influencing the allocation of healthcare resources, regulations for professional training and access to treatment (e.g., Barkham et al., 2010). For the purpose of this editorial, we will use the terms 'psychological therapy' and 'psychotherapy' synonymously. They are tools used to provide treatment in a clinical context to clinical groups.

Interestingly, proposals for the definition of psychological therapies are less frequent than expected. Hodges et al. (2011) summarized reviews for definitions and concluded: “Surprisingly, we were not able to find an explicit definition of what constituted a ‘psychological’ intervention in any of the reviews”. Although there are some attempts to define psychological therapy (see examples in the Supplement, e.g., Tolin et al., 2025), many of them show some weaknesses, are overly complex, not suitable for legal purposes, or are not feasible for a clear definition of boundaries.

In many countries, the definition of psychotherapy was based on the approval of a few general approaches (“schools”) of psychotherapy (often a selection of cognitive behavioral therapy [CBT], psychodynamic treatments, sometimes also family-systemic interventions, or person-centered interventions). The approval of psychotherapeutic schools and traditions can be more restrictive (e.g., CBT, psychodynamic and interpersonal psychotherapy [IPT] in Sweden; www.socialstyrelsen.se), more liberal (e.g., 23 treatment approaches in Austria; www.psychotherapie.at/patientinnen/ueber-psychotherapie), with very different concepts about “evidence based”, and different implications for public funding. Some experts criticize the strong orientation on traditional psychotherapy schools as “pre-scientific”, and argue in favor of more general and transtheoretical concepts of psychotherapy (Goldfried, 2020). Other definitions are based on an understanding of a psychotherapeutic situation as a one-on-one in-person setting, which challenges the inclusion of some modern developments such as videotherapy, blended therapy or even group therapy. Furthermore, psychological therapy as an overall scientific and clinical construct emphasizes a commitment to dialogue across different psychological therapies and the involvement of networks of scientists and practitioners (e.g., Castonguay et al., 2019). This requires a process of exchange and dynamic developments across professions and orientations, instead of establishing different schools as parallel structures (Goldfried et al., 2019). In the pursuit of personalized, evidence-based healthcare, a focus on few treatment traditions can hinder dynamic developments and the implementation of new, evidence-based approaches. For example, a recent survey showed that psychotherapists hold several distinct prejudices towards those from other theoretical orientations, which makes cooperation in research and clinical practice difficult (Schröder et al., 2026). This also leads to fragmentation within the field. Furthermore, many modern developments in psychotherapy do not adhere to the categories of a few traditional schools, but instead integrate treatment procedures from different traditions (e.g., schema therapy, mentalization-based therapy MBT or CBASP) or describe innovative approaches that are evidence-based with an own theoretical framework beyond the traditional schools. In many settings, treatments with different theoretical backgrounds are integrated into one comprehensive treatment program (Bohus et al., 2012; Venturo-Conerly et al., 2023). Some new developments, such as transtheoretical approaches (Lutz & Rief, 2024), process-based psychotherapy (PBT; Hofmann & Hayes, 2019, 2024), competence-based psychotherapy (Rief, 2021), or modular, mechanism-based psychotherapy (Herpertz & Schramm, 2022; Schramm et al., 2024) aim to overcome the traditional boundaries between theoretical frameworks. Cuijpers et al. (2025) outline that improvement of mental healthcare for depression needs to be done by multiple, incremental innovations, which requires an open and flexible system. Many of these innovations do not result from changes within specific school theories, but are motivated by better respecting other processes and context factors (e.g., societal and cultural factors; Wainberg et al., 2026).

These are aspects indicating the need for an umbrella definition of psychotherapy that is not based on single traditions, but that encompasses all interventions based on scientific principles including testable theories and empirical support for their effect. Furthermore, the definition should be aligned with the development of a measurement-based approach to psychological therapy (e.g., Delgadillo & Lutz, 2020). It should not be limited to a few theories, but should allow the consideration of all relevant theoretical and empirical approaches of modern psychological science and neuroscience. This definition should also facilitate innovation and help develop a psychotherapeutic system that allows for optimal personalization, or 'precision psychotherapy'.

## The German Interdisciplinary Group of Psychotherapists

In Germany, we have assembled a group of psychotherapists to develop an inclusive definition of psychotherapy rooted in scientific principles. The group was supposed to comprise people with different therapeutic backgrounds (CBT, psychodynamic, systemic therapy), experts from various disciplines (psychology, psychiatry, psychosomatic medicine) and age foci (adults as well as youth), and individuals with lived experience of psychotherapy, as well as early career clinicians. The group (which is identical to the authors of this editorial) screened existing definitions, and discussed the aspects that should be covered in a modern definition of psychotherapy. In contrast to other definition proposals (see Supplementary Table 1), this definition should be suitable for legal purposes, and should focus on the relevance of psychotherapy in health care systems; therefore, a focus on prevention and treatment of clinical conditions was assumed.

The group agreed that a definition of psychotherapy should include at least four components:

1. What are treatment means, 'tools'? (Modality of treatment)
2. How does the treatment work? (Proposed change mechanisms; assumptions on causal and evidence-based processes and concepts)
3. Who is the receiver of psychotherapy? (Target group)
4. Who provides psychotherapy? (Provider)

Having defined these categories, we propose the definition shown in Table 1. This should not be considered a final definition suitable for all purposes, but rather a basis for discussion and national adaptations. In the following section, we will discuss the proposal in more detail, summarizing the reasons behind it and exploring potential variations.

**Table 1:** Proposal for a Definition of Psychotherapy

**Category**	*Psychotherapy is*
Modality	*the evidence-based application of psychological procedures, *
Mechanisms	*based on scientifically sound assumptions about psychological processes*
Target	*to classify, diagnose, prevent, treat, and rehabilitate clinically relevant syndromes, disorders, and illnesses*
By whom?	*by specifically educated and trained people.*

Ad 1) **Modality**: Psychotherapy is the evidence-based application of psychological procedures: this defines the tool used by psychotherapists. While other clinicians may use pharmacological, surgical, electrostimulation or other procedures, psychotherapy is characterized by the systematic use of psychological procedures. 'Psychological' is not intended as a description of professions, but as a description of the intervention modality.

Ad 2) **Mechanisms**: Psychotherapy is based on scientifically sound assumptions about psychological processes relevant for problem definitions and change. This part emphasizes that professional psychotherapists should apply existing scientific knowledge about psychological processes not only originating from one single theory, but from the whole richness of knowledge and theories of empirical psychology, neuroscience and psychotherapy research. It also specifies that merely demonstrating positive pre- and post-intervention effects is insufficient; professional psychotherapy must be grounded in the best current knowledge of the underlying processes, theories of disorders and changes (Borsboom et al., 2021). Of note, while psychological therapy necessarily requires the definition of relevant psychological mechanisms, this should also be embedded in a broad biopsychosocial understanding of disorders and change processes.

Ad 3) **Target Group:** When defining the scope of psychotherapy, most people primarily consider mental and behavioral disorders as target groups of psychological interventions. Indeed, psychotherapy has become the recommended first-line treatment for most of these conditions (as single treatment or in combination with other treatments) (Rief et al., 2024). However, psychotherapy is also indicated for other conditions that are not classified as mental or behavioral disorders, but can be found under other categories of classification systems. Examples include insomnia and chronic pain conditions, with psychological therapies being among the first line treatments, although the main treatment focus can be on physical functioning. Psychotherapy is further indicated for several medical conditions typically considered “biomedical”, ranging from behavioral change programs to illness coping in diseases such as diabetes, cardiovascular conditions, or functional somatic disorders. Therefore, many clinically relevant syndromes, disorders and illnesses are subject to psychotherapy. Nevertheless, psychotherapy is not indicated for every medical problem and every patient; therefore, specifying that psychotherapy is an evidence-based application limits its use to clinical conditions for which there is evidence for the effect of applying psychotherapy. Thus, also sub-threshold conditions (e.g., high risk profile for psychosis) can justify psychological interventions.

Ad 4) **By whom?** Psychotherapy is provided by people who have been educated and trained to do so. In many countries, this is further specified by legal acts, defining who is allowed to provide psychological treatments in the health care system. In Germany, this is now mainly limited to psychologists and physicians who have received training in psychotherapy as part of their university education, and who have undergone further post-graduate training/ specialization. According to the suggested definition, psychotherapy can be delivered as videotherapy and also be blended with digital interventions. In contrast, task shared interventions can be delivered by not specifically trained psychotherapists (Karyotaki et al., 2022), e.g., under the supervision of approved psychotherapists. Such a definition also excludes mere digital interventions or AI based interventions (chatbots etc.) not embedded in a person-guided therapeutic process.

## Conclusion

In this editorial, we set out a framework for defining psychotherapy and make a proposal for a definition. We are aware that this is just a proposal and a starting point, and should be subject to further discussions. This definition is open to be modified according to national needs, future developments and other purposes, but we recommend considering the four criteria for definitions and adaptations. Our suggested definition is not bound to specific treatment orientations, but is intended to encompass traditional treatment approaches as well as new developments beyond these traditions. We aim to promote the evidence-based provision of psychological treatments in healthcare systems and legal acts at a national level that help provide this useful intervention to all patients in need of it.

## Supplementary Materials

The Supplementary Material includes examples of definitions of psychological treatments / psychotherapy (for access, see Rief et al., 2026S).



RiefW.
AsbrandJ.
BaumgärtnerL.
DingerU.
FlückigerC.
HeinrichsN.
HerpertzS. C.
LincolnT.
LipinskiS.
LutzW.
StraussB.
TaubnerS.
VorthmannO.
KleinJ. P.
 (2026S). Supplementary materials to "How to define psychological therapy and psychotherapy? – An interdisciplinary proposal"
[Examples of definitions of psychological treatments / psychotherapy]. PsychOpen. 10.23668/psycharchives.21895
42632823
